# Supplementary material for: Content-rich biological network constructed by mining PubMed abstracts
Source: BMC Bioinformatics. 2004 Oct 8;5:147. doi: 10.1186/1471-2105-5-147 (PMC528731; doi:10.1186/1471-2105-5-147)
Supplement: Additional File 2 — The original results of the above study (non-essential files are deleted to keep the file size under the limit set by BMC bioinformatics). [file 1471-2105-5-147-S2.bz2 › chilibotAdditionalFile2/dip05/601ID10052460E10980/html/HOXB1_PBX1.html]

 


 **HOXB1** and **PBX1** 
  
Found 178 abstracts in PubMed, retrieved 05.  
 

 What does Google say? 
 PDF only 
| .edu only 

---

**Interactive relationship** (e.g. stimulation, inhibition, etc)

**Stimulatory relationship**- However, on the  **hoxb1**  autoregulatory enhancer, Hoxa1 and Hoxa1 NA KR may display distinct activity despite the presence of Pbx  [ **PBX1** ] , in a cell type dependent manner.  Ref: 12060683 Nucleic Acids Res, 2002
**Neutral relationship**

**Non-interactive relationship** (e.g. studied together, co-existance, homology, etc.)

- We show that, in P19 embryonal carcinoma cells, GCs potentiate RA induced expression of the murine Hoxb 1  [ **HOXB1** ]  gene through an autoregulatory element, b1 ARE, recognized by the  **Pbx1**  and  **HOXB1**  homoeodomain proteins.  Ref: 12487626 Biochem J, 2003
- Therefore, we hypothesize that expression of two additional mesenchymally derived nuclear transcription factors, retinoid X receptor alpha RXR alpha and homeobox  [ **PBX1** ]  b 5 Hoxb  [ **HOXB1** ]  5, would also be altered in hypoplastic lungs.  Ref: 12505043 J Surg Res, 2002
- Additional transfection experiments showed that the  **Pbx1**   **HOXB1**  heterodimer was the target for the GC effect.  Ref: 12487626 Biochem J, 2003
